# Early experience on a modern, thin cochlear implant family. A retrospective, international multicenter study

**Published:** 2018

**Authors:** A Perenyi, F Toth, AA Nagy, J Skrivan, J Boucek, DC Gheorghe, A Neagos, JG Kiss, J Jori, L Rovo

**Affiliations:** * University of Szeged, Albert Szent-Györgyi Clinical Center, Department of Otorhinolaryngology, Head and Neck Surgery, Szeged; ** Department of Otorhinolaryngology, Head and Neck Surgery, Charles University in Prague, Second Faculty of Medicine, Motol University Hospital; *** Department of Otorhinolaryngology, Head and Neck Surgery, Charles University in Prague, First Faculty of Medicine, Motol University Hospital; **** ENT Clinic “Maria Sklodowska Curie” Hospital, “Carol Davila” University of Medicine and Pharmacy, Bucharest; ***** Emergency County University Hospital of Târgu Mures, ENT Department

**Keywords:** deafness, cochlear implant, minimally invasive surgery, subperiosteal pocket, fixation

## Abstract

Rationale: Cochlear implantation is the most effective method of rehabilitation for patients with severe to profound sensorineural hearing loss. Binaural hearing forms the basis of the development of hearing-associated cortical networks in infants and toddlers, but simultaneous bilateral implantation is often postponed due to the demands of classical surgical methods, which are associated with large incisions and a deep bony well. Objective: The authors report on the use of a modern, thin implant type and the possibilities it provided to simplify the surgical technique. Methods and results: Recent models of the Cochlear™ Nucleus® implant family were studied in an international retrospective multi-center study: 6 otolaryngologists in 5 centers shared their experiences on 73 consecutively implanted, thin implants. The surgical incision could be made shorter than before and only shallow bony wells or none at all were created in 4 out of 5 centers. No complications occurred. Discussion: This study underlines that implants with thin electronics capsules enable a simplified, fast and safe implantation procedure that allows simultaneous bilateral cochlear implantation.

## Introduction

Early and successful audiological rehabilitation of infants and toddlers is of the utmost importance because the untreated severe hearing loss would impede their speech abilities [**1**][**2**]. If high-power hearing aids do not bring enough benefit, cochlear implantation is indicated. Our centers’ recommendation is that cochlear implantation should be performed before the age of 18 to 24 months because the connectome [**3**] (a network of effective synaptic connections and neural projections) continues to mature up until this age [**4**][**5**]. Later, the plasticity of the human brain gradually diminishes. In practice, this means that if cochlear implantation is performed in time, the toddlers have a high chance of reaching equivalent levels of speech performance to their normal-hearing peers without much delay in their speech development [**6**][**7**]. Early rehabilitation may be delayed not only by late diagnosis of deafness, but also by the surgical requirements of the classical technique for cochlear implantation (large access, risk of complications, long surgical time, blood loss that are problematic aspects most commonly in infants and toddlers) for which reason the use of thinner implants is advantageous. The surgery is associated with several risk factors, predominantly in infants and toddlers because the bone and soft tissues are very thin in these age groups [**8**], which makes subjects more prone to complications [**9**-**12**].

The classical surgical technique for cochlear implantation, dating back some decades, required large access, which was not a major problem whilst the majority of the implantations were performed on adult subjects. Today, however, a large number of surgeries are performed on infants and toddlers. The earlier types of cochlear implants were thick in order to provide good impact resistance, and for this reason, a bony well to sink the implant into the skull was required. This sometimes resulted in bulging soft tissues, which were associated with discomfort during sleep and for those wearing glasses and increased risk of soft tissue necrosis over the implant. Creation of a bony well required a long, up to 10 to 15 cm incision and wide access [**13**] that considerably compromised the stability of the soft tissues. Consequently, in order to prevent dislocation of the implant electronics, the surgeon had to fixate the implant package to the bone with non-absorbable sutures, mesh or screws [**14**-**16**]. The bony well, with a sharp rim, contributes towards the prevention of implant migration. This classical method, however, is associated with several hazards, especially in infants and toddlers, because they have very thin bone and soft tissues at the implant site [**6**]. Potential severe complications are cerebral infarction [**9**], epidural hemorrhage [**10**], infarction of temporal lobe [**11**], lateral sinus thrombosis [**11**], subdural hemorrhage, liquorrhoea [**12**], and soft tissue necrosis. If a bony well is created, the skull will be weakened and will have lower ability to resist mechanical injuries. Furthermore, the bony well requires a large view and incision and the overlying soft tissues will push the implant less tightly to the skull. A longer incision is associated with a longer surgical time, more blood loss, more time needed for coagulation and wound closure and longer hospital stays and postoperative care [**17**].

The subperiosteal pocket technique has become a widely used method of cochlear implantation: Balkany et al. used the tight temporal pocket and further tightened the pocket with periosteal sutures to fixate the implant [**16**], while Jethanamest et al. used the “subperiosteal tight pocket” without any other fixation of the implant [**19**]. Recently, Turanoglu et al. reported their finding that the internal electrical unit of the implant device, implanted with the subperiosteal temporal pocket technique, fixates itself by causing bone remodeling and making an impression on the skull [**20**]. Regarding displacement of the implant, no difference was found between the classical and the tight subperiosteal pocket technique so far [**21**][**22**].

To optimize outcomes, it is advised to aim for an early diagnosis, bilateral auditory rehabilitation and quick, minimally invasive and safe cochlear implant surgery. When indicated, simultaneous bilateral implantation is recommended because sequential procedures are linked with two courses of anesthesia, double surgical load and double the hospital stay. However, the disadvantages of surgery as discussed above mean that bilateral implantation in one surgical procedure is often not possible. The leading cochlear implant manufacturers are familiar with these dilemmas and develop their devices based on consensus with surgeons. A critical aspect of the design of the device is size. Decreasing size, especially thickness, facilitates minimally invasive surgical techniques as thin implants can be implanted either into a shallow bony well or even without a bony well [**23**][**24**]. Although other manufacturers have aimed to decrease the thickness of their cochlear implants, the most conspicuous change can be observed across the Cochlear™ Nucleus® implant series. The newest Profile devices from Cochlear Ltd. (Sydney, Australia) have an almost 50% decrease in thickness compared to Nucleus® Freedom devices. This provides an opportunity to examine the practical value of such changes and allows us to assess the first experiences with the device from the user’s perspective.

The aim of our study was to assess the advantages of this low-profile implant family with regards to the modification of the surgical technique and any changes in comparison with the earlier type of implants. Experienced surgeons were interviewed and given a self-administered questionnaire. They were asked: Does the design of the implant allow modification of the classical surgical technique to allow a simpler and safer procedure?

## Methods

**Fig. 1:**
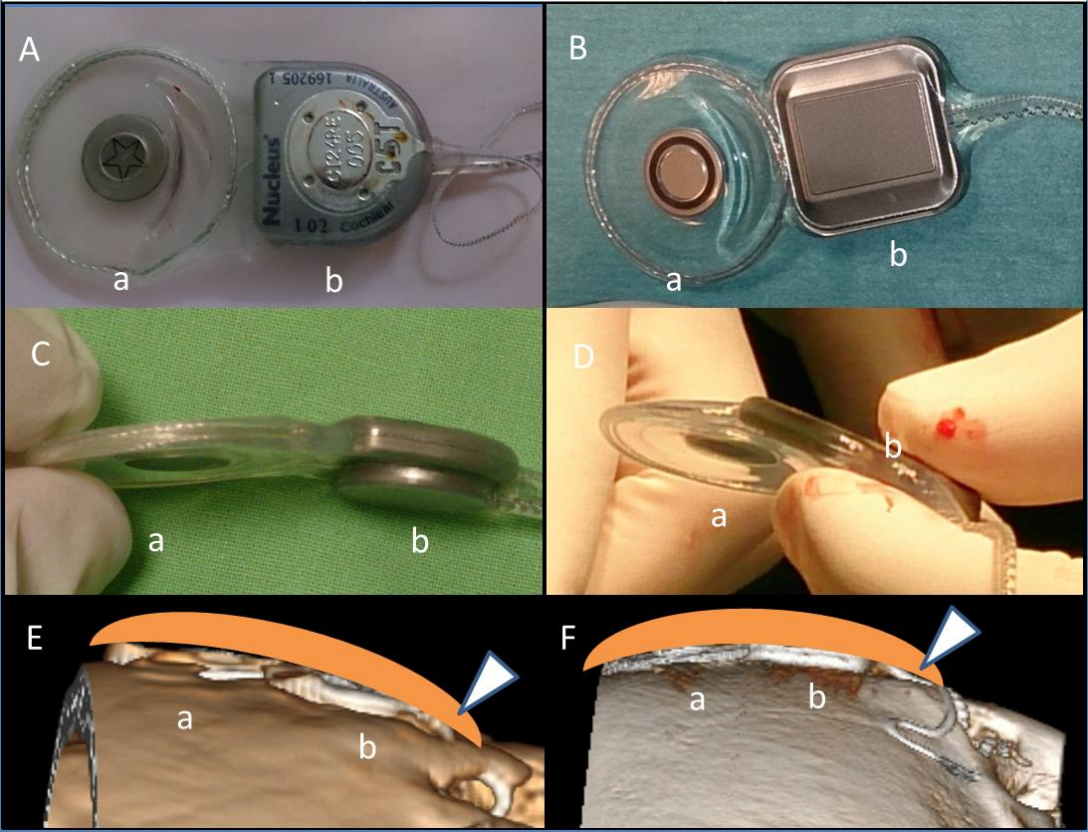
Cochlear™ Nucleus® Freedom (on the left) and Cochlear™ Nucleus® Profile (on the right) implants from above (A and B) and side view (C and D). The dimensions are detailed in the table. E and F show the three dimensional CT reconstructions (software: RadiAnt DICOM Viewer). The electronics capsule (a) and the antenna (b), in the middle of which a magnet is mounted, can be easily recognized. The arrowhead represents the soft tissue over the implant. The profile of the implant protrudes out of the bony surface only as much as the earlier type of implant.

The surgeries were performed using the Cochlear™ Nucleus® Profile implants (CI512 and CI522). Nucleus® CI532 that was marketed later has the same low-profile electronics capsule, thus the results can be projected to all three implant types within the Profile family. The profile thickness of the implant was decreased compared with the earlier model, the Nucleus® Freedom, from 6.9 mm to 3.9 mm. Although the implant electronics capsule changed in shape, the rounded edges became more angular, the overall dimensions did not change significantly (**Figure 1**).

**Figure:**
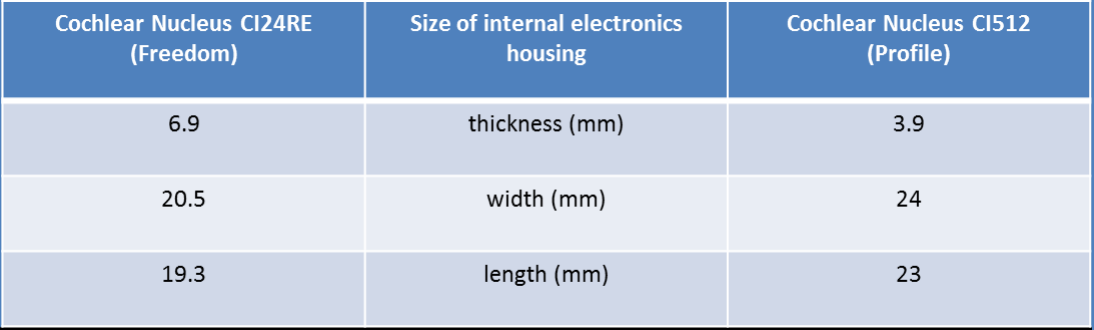


**International multi-center retrospective study**

The surgical experiences with the thin implant types were collected and compared in a retrospective multi-center study. The surgeons gave feedback about the changes in their surgical technique with the thin implant family compared with the earlier, thicker types.

Five leading cochlear implant centers in Central and Eastern Europe were enrolled: **1.** The University of Szeged, Albert Szent-Györgyi Clinical Center, Department of Otorhinolaryngology, Head and Neck Surgery, Szeged, Hungary (21 Profile implants) **2.** Department of Otorhinolaryngology, Head and Neck Surgery Charles University in Prague, Second Faculty of Medicine Motol University Hospital, Prague, Czech Republic (18 Profile implants) **3.** Department of Otorhinolaryngology, Head and Neck Surgery Charles University in Prague, First Faculty of Medicine Motol University Hospital, Prague, Czech Republic (11 Profile implants) **4.** ENT Clinic “Maria Sklodowska Curie” Hospital, “Carol Davila” University for Medicine and Pharmacy, Bucharest, Romania (17 Profile implants) **5.** Emergency County University Hospital of Targu Mures, ENT Department, Targu Mures, Romania (6 Profile implants)

**Fig. 2:**
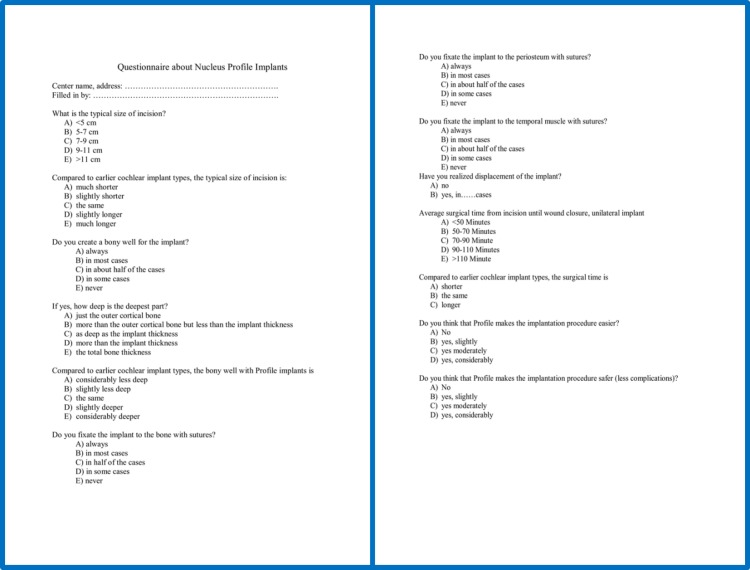
The English language self-administered questionnaire

Six experienced otolaryngologists (the average years of experience with ear surgery was 26 years) were asked to fill in a self-administered questionnaire regarding their opinion about the thin implants. The official language of the questionnaire was English (**Figure 2**).

The surgeons provided answers based on their subjective surgical experiences and the documentation collected about the implant surgery. A total of 73 thin implants were implanted into 59 recipients, up to 31 January 2015. The recipients were children and adults with an average age of the recipients between 17-23 years. Sex ratio was 30 females and 29 males. Bilateral implantation was performed in 14 subjects.

The First Faculty and Second Faculty of Medicine Motol University Hospital acted as independent centers. The following topics were covered: **1.** The length of the surgical incision and its relation to the earlier type of implant **2.** Necessity and dimensions of a bony well for the implant electronics **3.** Fixation of the implant electronics with sutures **4.** The incidence of intraoperative and early postoperative complications **5.** Dislocation of the implant **6.** Change in surgical time **7.** The opinion of the surgeon if implantation of the thin implants is easier or safer **8.** The general opinion of the surgeon and comments

## Results

The length of the surgical incision measured was less than 5 cm in 2 centers and between 5 and 7 cm in 3 centers. When compared with the earlier type of implant, the incision was found to be considerably shorter (3 out of 5 centers; 60%), slightly shorter (1 out of 5 centers; 20%), and unchanged (1 out of 5 centers; 20%).

A bony well was created only in a few cases (2 out of 27 implants) in 2 centers and in all cases in 3 centers (46 implants). If a bony well was created, it was performed by removing the outer cortical bone only in three centers. The bony well was deeper than the outer cortical bone but less deep than the implant thickness in one center and the bony well was as deep as the implant thickness in one center. When compared with the earlier type of implant, the bony well was reported to be considerably shallower in 4 centers and slightly shallower in 1 center.

None of the centers fixated the implant to the bone. Periosteum and temporalis muscle was always used for fixation in 2 out of 5 centers. No intraoperative or early postoperative complication was reported by any of the centers.

Minimal displacement of implant electronics was registered in one center, without the need for revision surgery. This center reported having drilled a bony well as deep as the implant thickness and used the periosteum for fixation.

The typical surgical time for unilateral surgery from incision until wound closure was reported to be less than 50 minutes in 2 centers, 50 to 70 minutes in 1 center and 90 to 110 minutes in 2 centers, while surgery with the thicker implants took approximately 10 to 25 minutes longer. The shorter times were seen in those centers in which a bony well was not drilled. When compared with the earlier types of implants, the surgical time was shorter in all 5 centers.

The surgeons found the implantation procedure for the recent type of implant considerably easier in 2 centers, moderately easier in 1 center and slightly easier in 2 centers.

They also found the implantation considerably safer in 2 centers, moderately safer in 1 center and slightly safer in 2 centers.

The surgeons all agreed that the thin nature of the new implant did not necessitate a classical bony well.

## Discussion

Universal newborn hearing screening enables early detection of hearing loss in infants and their early audiological rehabilitation, even under the age of 1 year. Binaural hearing not only helps to localize the source of the sound but also improves speech perception, especially in noisy environments [**25**-**27**]. Therefore, optimum outcomes can be achieved in these infants by providing them with bilateral cochlear implants.

Although several factors are in favor of simultaneous bilateral implantation [**28**][**29**], this procedure is associated with longer surgical time and increased load, especially in infants and toddlers. For this reason, an important goal of implant surgery is to use faster and more minimally invasive surgical techniques. In order to prevent bulging of soft tissues over the thicker implants, the classical technique of cochlear implantation requires the drilling of a bony bed and fixation of the device to the skull and is associated with several risk factors, predominantly in infants and toddlers. The bone and soft tissues are very thin in these age groups [**8**] which make subjects more prone to complications [**9**-**12**]. The biggest advantage of thin implants is that they can be implanted into a shallow bony well or even without a bony well and often without the need for fixation.

The surgeons participating in the study all agreed that the thin nature of the new implant did not necessitate a classical bony well and that the reduction in drilling definitely decreased the risk of a dural injury, with a further advantage that the implant was less bulging under the skin. Nevertheless, for those who decided to use a bony well, this could be easily created because in their opinion an anterior rim sufficed to prevent migration of the implant.

**Fig. 3:**
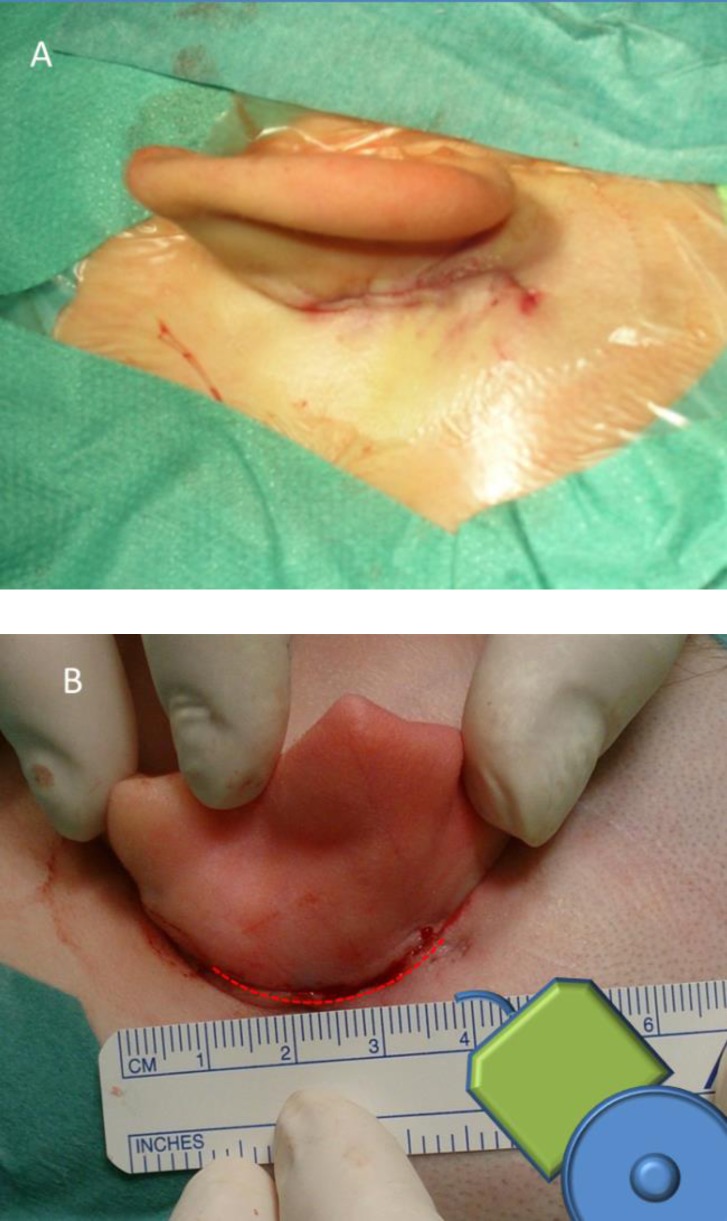
Two examples of the shortest incisions. A) A short, linear incision with superior-posterior extension. B) A short, curved incision in the retroauricular fold. The drawing indicates the approximate location of the implant.

The results of the multi-center questionnaire show that the surgical techniques made possible by the thin implants had several benefits compared to the older devices, which were clinically relevant: **1.** The surgical incision used was shorter, which is associated with less blood loss, as the bleeding stops sooner and wound closure is faster (**Figure 3**).

**2.** No bony well was necessary, or if used it could be made shallow. This reduces the risk of complications and time can be saved (Figure 5). **3.** No other fixation of the implant to the skull other than the subperiosteal tight pocket has to be used. This ensures enough stability of the implanted device and time can be saved. **4.** The rate of displacement (1 out of 73 cases) was comparable to other techniques. Note: The only displacement was observed at the site in which the standard technique is to make a bony well as deep as the implant thickness. A deep well requires a large view and the tissues of the subperiosteal pocket will be weakened.

**Fig. 4:**
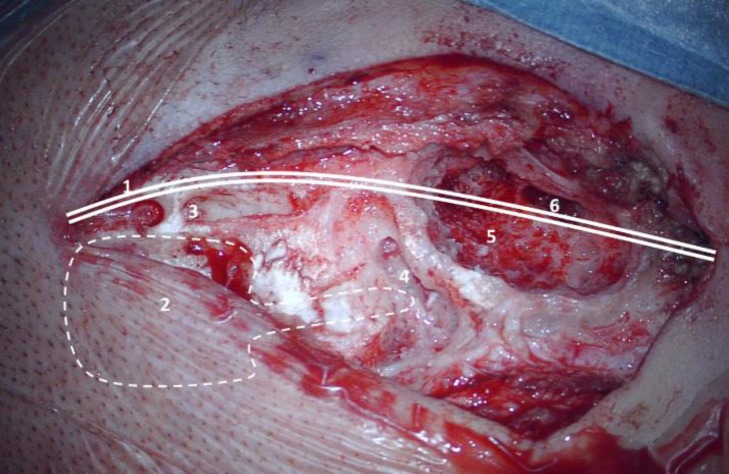
An example of sinking the implant into a bony well: Meticulous and time-consuming surgical steps may be required. Sinking the implant into a bony well requires several surgical steps. 1: Long incision and large exposition of the skull. 2: The outline of the bony well. 3: A bony tunnel in which the fixation sutures are inserted. 4: A bony tunnel in which the stimulating electrode is inserted. 5: A limited mastoidectomy cavity. 6: Posterior tympanotomy. Steps 1-4 can be omitted if thin implants are used.

**5.** The above-mentioned factors made the implantation procedure simpler, reduced surgical time and decreased the load of postoperative care and duration of hospital stay. This is a considerable advantage, especially in simultaneous bilateral implantations in infants and toddlers.

## Conclusion

Thin implants are advantageous because they fit the shape of the skull and cause only slight protrusion of the soft tissues, even without a bony well. The smaller incision (exploration) is associated with less blood loss and a drop in surgical time.

Our study underlines that the new thin implants are associated with real benefits, in that they allow us to further develop and simplify the surgical technique for cochlear implantation. One should note that if the surgical incision is larger than necessary and the integrity of the soft tissues is not preserved, the pocket for the implant will be unnecessarily weakened. In such cases, in order to prevent implant displacement, the implant should be fixated to the bone (bony well, sutures).

In the authors’ opinion, the development of cochlear implant design should be directed towards a decrease in thickness and adaption to the shape of the skull.

## Conflict of Interest

The authors declare that there is no conflict of interest.
